# Visual Function of Egyptian Children with Low Vision and the Demographic Determinants

**DOI:** 10.4103/0974-9233.61222

**Published:** 2010

**Authors:** Boshra Mohammed El Byoumi, Ahmed Mousa

**Affiliations:** Department of Ophthalmology, The Memorial Institute for Ophthalmic Research, Al Ahram Street-Giza-Egypt; 1 Department of Ophthalmology, College of Medicine, King Saud University, Riyadh, KSA

**Keywords:** Children, Low Vision, Quality-of-Life

## Abstract

**Aims:**

To determine whether the LV Prasad-Functional Vision Questionnaire (LVP-FVQ) could be used to assess self-reported visual function and quality of visual life in Egyptian school aged children.

**Materials and Methods:**

The LVP-FVQ was used to assess the quality of visual function in school-age children. All subjects were students at the time of assessment. Subjects underwent a visual function assessment that included distance and near visual acuity, contrast sensitivity, color vision and visual field examination where possible. Data analysis were for both descriptive and inference statistics. A *P* < 0.05 was considered statistically significant.

**Results:**

Fifty children aged 11.28 ± 3.5 years (range, 5 years to 18 years) with moderate-to-severe visual impairment most of their lives were enrolled. Twenty-two subjects (44%) had albinism, 18 (36%) subjects had hereditary retinal dystrophy, 6 (12%) subjects had cone dystrophy, 2 (4%) subjects had bilateral amblyopia and 2 (4%) subjects had congenital coloboma without other disabilities. The four most difficult tasks were related to the following daily activities alluded to in the questionnaire such as reading a textbook at arms length, copying from the blackboard, seeing somebody across the road and identifying colors. There was no statistically significant association between the demographic variables and the level of visual functioning, sex, age, type of school, family history or consanguinity (*P* > 0.05 for all variables).

**Conclusion:**

LVP-FVQ can be used to screen Egyptian children with visual impairment. Input and integration of the parents and the school teachers to evaluate the child s behavior at home and the school is essential to developing a balanced questionnaire.

## INTRODUCTION

Identifying and studying visual function in visually impaired children younger than 15 years of age is challenging and is based on the ability of the child to comprehend the testing procedure and the ability of the child to cooperate with the test tool.^1^,^2^ Additionally the sensitivity, specificity and predictive values of different tests and results obtained after screening by various allied health personnel needs consideration. For example, less than optimal rates of identification of visual impairment for different age groups, have been reported even when screening was performed by trained paramedics including optometrists.^3^

In Egypt, population-based assessments of the impact of visual impairment on visual function have not been previously studied. It is well known that subjective complaints of difficulty do not necessarily correlate well with objective measurements.^4^,^5^ Difficulties with spatial tasks do not appear to be related to the level of visual acuity and visual field loss.^6^,^7^ Performance may depend on an individual's ability to localize objects in the surrounding visual world.^8^,^9^ High contrast Snellen acuity measurements do not necessarily reflect daily visual function and health status. Therefore, the development of a questionnaire for the assessment of functional vision in children would be valuable. Children with visual impairment often cannot or do not express their problems. Moreover, the activities of children vary with age, and it is therefore difficult to develop a single instrument that can serve as a measure of children's functional problems.^10^

Gothwal *et al*.^11^ developed a questionnaire to assess the self-reported functional abilities of visually impaired children: The LV Prasad-Functional Vision Questionnaire (LVP-FVQ). It uses simple and direct questions about the daily activities of the children which can be used by allied health care professionals such as social workers to record vision problems. In the present study we evaluated the LVP-FVQ as an adjunct to clinical measures in assessing self-reported functional vision and to test the validity of LVP-FVQ as a reliable tool for cross-cultural use, in this case on a Egyptian children.

## MATERIALS AND METHODS

Sixty five children with visual impairment who were referred to the low vision clinic at the Memorial Institute for Ophthalmic Research (Giza, Egypt) were assessed in this study. All subjects were classified as visually impaired using the World Health Organization criteria for persons with low vision.^12^ The first author, a Low Vision Consultant personally administered all questionnaires. A quality check on the data was performed by correlating the responses to clinical evaluation for every subject. For example a child may have responded that the color vision is normal yet clinical examination was positive for color blindness. Subjects with other impairments such as hearing loss or intellectual impairment were excluded from the study. The data were also evaluated according to inclusion criteria and subjects were not identified in any manner for statistical analyses. This study adhered to the tenets of the Declaration of Helsinki. For example, we ensured that children who could correctly understand and respond to the questionnaire were only included. Missing, incomplete, or conflicting data were investigated in every instance to determine a cause and rectified where appropriate. Based on the data quality checks, a total of 50 subjects were selected for the data analysis process.

### Questionnaire

The LVP-FVQ was translated into Arabic by the first author and was used for assessment of quality-of-life of the subjects. The questionnaire uses 19 items (questions) designed to cover four main aspects of vision: Distance vision (six questions), near vision (six questions), color vision (two questions) and visual field (five questions). These 19 items are directly related to difficulties in performing a variety of tasks that mainly rely on visual capabilities. An additional item (question 20) was related to the global self-assessment of a subject's vision in comparison to his or her friends with normal vision.

In the LVP-VFQ reponses are based on a 5-point scale (0-4) where either ‘Yes’ or ‘No’ is first required for each question. If the answer was ‘No,’ it meant ‘No difficulty,’ the score was zero. If the answer was ‘Yes’, then the level of difficulty was categorized into 1 to 4 categories (1 meant ‘a little difficult’ whereas 4 denoted ‘unable to do the activity due to visual reasons’). All items were scored in the same fashion using this scale. ‘Not applicable’ was used, instances that were gender specific such as threading a needle or lacing up shoes. Measurement of visual acuity was performed with the Lighthouse distance chart for low vision (Lighthouse International, New York, NY, USA) and near vision was evaluated with the LEA symbol chart (Good Lite Co., Elgin, Il, USA) at a distance of 20 cm.

In addition to an ophthalmic evaluation, other visual function tests were conducted, including color vision test, contrast sensitivity and visual field testing. Additional investigations were performed for diagnosis of retinal disorders included electroretinography (ERG), electrooculography (EOG) and visual-evoked potential (VEP). A customized database using Microsoft Access 7 (Microsoft Corp., Redmond, WA, USA) was designed to collect data for this study, which was entered by ophthalmic staff and reviewed by the authors.

The total raw score was derived for each subject and considered a discrete variable (and used as an indicator in the analysis). Descriptive analyses were performed on demographic variables and the total raw score. The *Chi*-square test was used to assess the relationship with each level of severity in visual functioning. SPSS 16 (SPSS Inc., Chicago, Ill, USA) was used for all statistical analyses.

## RESULTS

**Figure 1:**
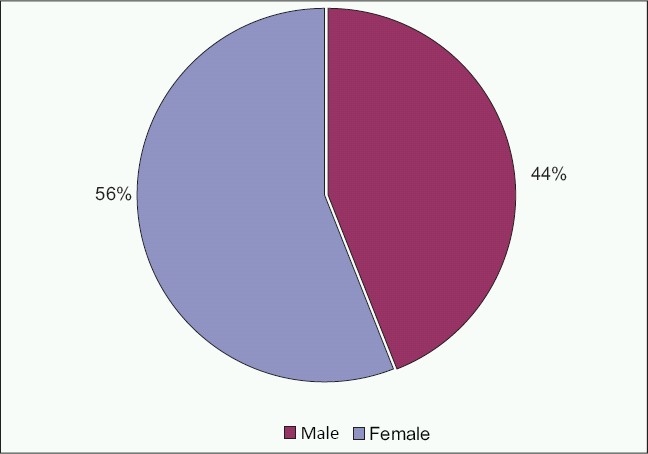
Sample distribution by sex

The study included 50 school-age children who were visually impaired since birth (eg. due to albinism) or a few years after birth (eg. due to cone dystrophy).The mean age of the cohort was 11.28 ± 3.5 years, (range, 5 years to 18 years) which was comprised of 28 (56%) females and 22 (44%) males [Figure 1].

In this cohort, the majority of subjects were visually impaired due to hereditary retinal dystrophy. Of these, 2 subjects (4%) had retinitis pigmentosa, 6 subjects (12%) had Stargardt's disease, 10 subjects (20%) had vitelliform dystrophy, and 6 subjects (12%) had cone dystrophy. Other causes of visual impairment included 22 subjects (44%) with oculocutaneous albinism, 2 subjects (4%) with bilateral amblyopia, and 2 subjects (4%) with congenital coloboma.

Two subjects (4%) attended a school for the blind, 12 subjects (24%) attended a school for individuals with low vision and35 subjects (70%) attended regular schools [Figure 1].

**Figure 2:**
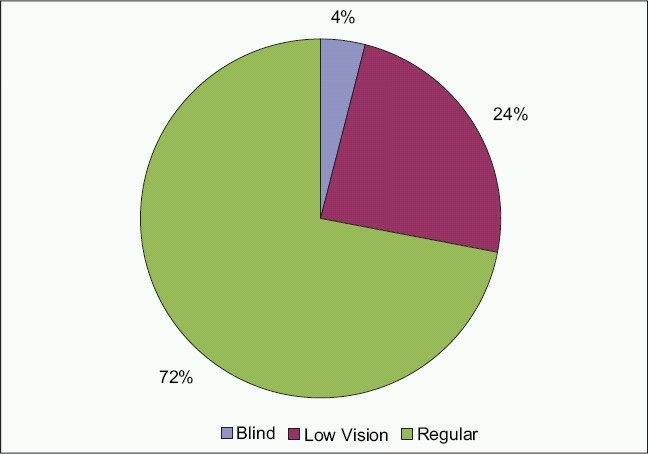
Sample distribution by school

**Figure 3:**
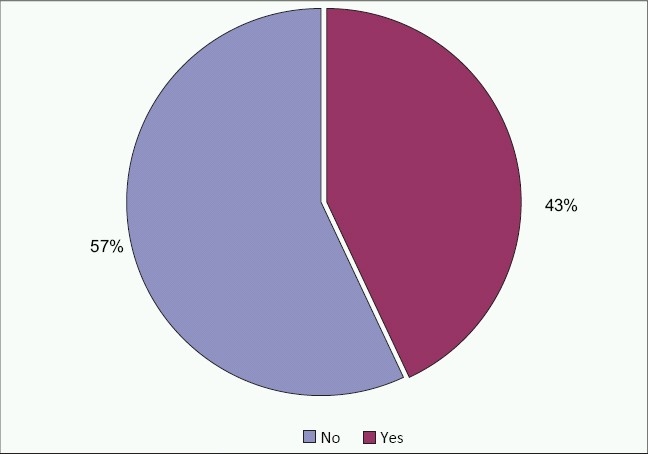
Sample distribution by family history

A family history of visual impairment was positive for 28 subjects (56%) and negative in 21 subjects (42%) [Figure 2]. Thirty nine subjects (78%) were products of a consanguineous marriage whereas 10 subjects (20%) were not [Figure 3].

**Figure 4:**
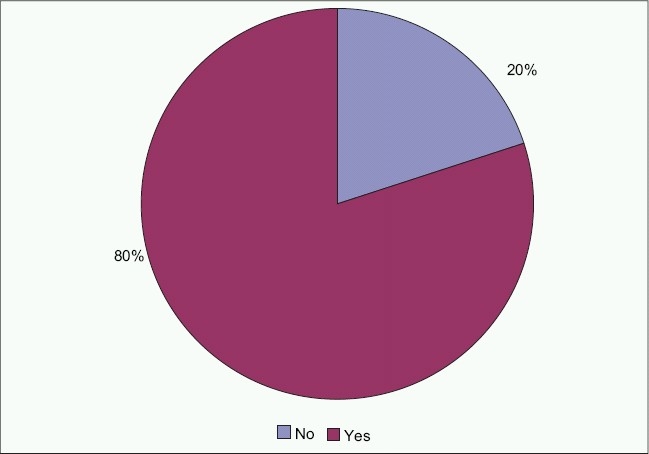
Sample distribution by parents being relatives

The mean raw score of the questionnaire data was 7.44 ± 11.01 (range, 0-46). The most frequently reported difficulties were reading a textbook at arm's length, copying from the blackboard, seeing somebody across the road and identifying colors [Table 1]. The probability of subjects using categories 1, 2 or 3 (that included: A little, moderate amount or a great deal) was very low for all values of person-item measure. Self-estimation of visual function was compatible with clinical findings in 11 subjects (22%), was underestimated by 26 subjects (52%) and overestimated by 13 subjects (26%) [Figure 4].

**Table 1:** The response pattern to the different questions in the LV prasad visual function questionnaire

Question	Score									
	0		1		2		3		4	
	No.	%	No.	%	No.	%	No.	%	No.	%
Q1	34	68	1	2	5	10	6	12	4	8
Q2	46	92	2	4	2	4	-	-	-	-
Q3	45	90	-	-	-	-	4	8	1	2
Q4	35	70	-	-	3	6	10	20	2	4
Q5	45	90	-	-	-	-	4	8	1	2
Q6	47	94	-	-	2	4	1	2	-	-
Q7	39	78	-	-	-	-	11	22	-	-
Q8	43	86	1	2	3	6	2	4	1	2
Q9	41	82	4	8	1	2	4	8	-	-
Q10	40	80	3	6	1	2	5	10	1	2
Q11	48	96	-	-	-	-	1	2	1	2
Q12	45	90	1	2	1	2	3	6	-	-
Q13	47	94	-	-	1	2	1	2	1	2
Q14	46	92	-	-	1	2	1	2	2	4
Q15	43	86	-	-	-	-	5	10	2	4
Q16	47	94	-	-	1	2	2	4	-	-
Q17	46	92	-	-	2	4	2	4	-	-
Q18	37	74	2	4	5	10	4	8	2	4
Q19	37	74	2	4	5	10	4	8	2	4

There was no statistically significant association between the demographic variables and the severity of visual dysfunction (*P >* 0.05) as well as the self-estimated level of visual functioning [*P > *0.05, Table 2].

**Table 2:** The relationship between the demographic variables and the level of severity of visual dysfunction and the self-estimated level of visual functioning

Demographic variables	Level of severity of visual impairment (*χ^2^*, *P* value)	Self-estimated level (*χ^2^*, *P* value)
Age	41.30, 0.80	15.17, 0.13
Sex	10.67, 0.058	2.21, 0.33
Type of school	11.17, 0.51	3.69, 0.45
Family history	7.97, 0.44	3.2, 0.20
Parents related (consanguineous marriage)	10.79, 0.29	3.73, 0.15

### Quality of life with gender

**Figure 5:**
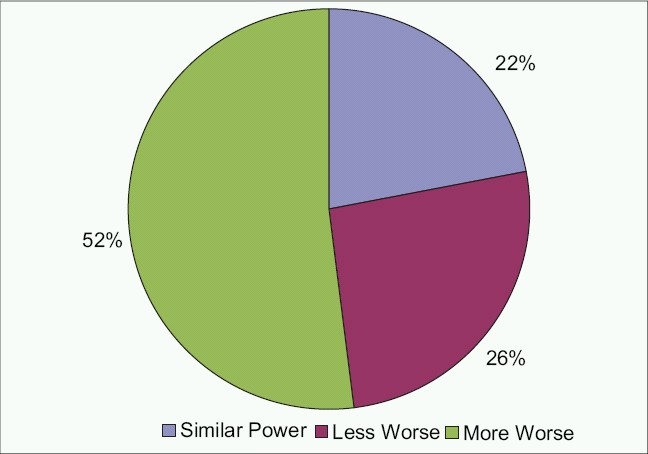
Distribution of self-estimation

Visual function scores were 10.1 ± 10.3 for males and 5.39 ± 11.3 for females. Despite double the difference in scores between males and females, statistically significance was not achieved [*P > *0.05, Figure 5].

## DISCUSSION

There are several internationally applicable instruments for measuring disability scores and quality of life in disabled indviduals. However most of these tools were designed and implemented for adults. There is a relative paucity of literature regarding similar tools for the pediatric ophthalmologist.^13^,^14^

The present study is the first of its kind to test the administration and effectiveness of the LVP-FVQ in visually impaired Egyptian children. The questionnaire was translated to Arabic and used to assess quality-of-life measures in 50 children. This study tested the main aspects of functional vision including a self-comparison to normally sighted peers.

In the study cohort, hereditary retinal conditions were the most common cause of visual impairment (48%). These results are similar to Gothwal *et al.*,^11^ who reported that the retinal disorders such as heredomacular degeneration and retinitis pigmentosa comprised 55% of the visual impairment in their cohort of 78 subjects.

In the present study, a number of subjects were visually impaired early in life and the results may reflect a biased self-estimation of difficulty resulting in underestimation or overestimation. A similar phenomenon was demonstrated by Wright *et al*. using a different tool.^15^ One method to address this drawback of self-estimation is to have the subjects actually perform the required tasks in the presence of a clinician or investigator.^16^ However in practicality the logistics of such an evaluation is tenous at best.

Age, sex, consanguinity, a positive family history of visual impairment, or type of school were not statistically significantly associated to the level of severity of visual function. Similarly the demographic variables and the self-estimated level of visual functioning were not statistically significantly associated. One explanation for the lack of association might be the small sample size and the level of visual impairment that ranged from moderate to severe.

We found that a child's comprehension was not dependent on age of the child but on the age at which the visual impairment was discovered. Gothwal *et al*.,^11^ reported that children between 5 to 7 years of age had difficulty comprehending the questions. Perhaps the difference in the type of subjects tested in our study compared to Gothwal *et al*.^11^ explain this discrepancy. In the Gothwal *et al*.^11^ study, 17.9% of subjects had one sighted eye, which may have influenced comprehension of the questionnaire among subjects.

The mean raw score was 7.44 ± 11.01 (range, 0-46). The most frequently reported difficulties were reading a textbook at arm's length, copying from the blackboard, seeing somebody across the road and identifying colors. In a study of visual impaired Indian children,^11^ the mean raw score was 210.5 ± 74.9 (range, 51 to 298) which was higher than that obtained in our study. The greater degree of visual impairment in our cohort explains the differences in the score. Of note was the wide range of scores seen in both studies. The four most difficult tasks were all related to visually demanding activities such as reading a textbook at arm's length, reading destination details of a bus, threading a needle, and copying from the blackboard despite sitting in the front row in class. The easiest tasks (those items that required the least visual ability) were daily living tasks such as applying paste to a toothbrush, locating food on a plate, walking alone in the corridor at school and walking back home at night, for which the subjects reported little or no difficulty. These outcomes are the same as a those reported for visually impaired Indian children.^11^

In the study by Gothwal *et al*.,^11^ the probability of subjects using categories 1, 2 or 3 was near zero for all values of person-item measure, which was similar to that observed in our study. Gothwal *et al*.^11^ suggest the cause was that the children found it difficult to remember the four response categories. Additionally, children may have not been able to make accurate judgments of scale i.e., they could either perform the task or they could not, and if they could, they did not have a basis for comparison to mild, moderate or great difficulty because they always perform the task in one particular manner. However, we believe this outcome occurred because most children were visual impaired since birth or in early childhood and therefore could not judge the level of severity.

The authors of the LVP-FVQ suggested that the results can be used to advise the parents and teachers of visually impaired children on simple interventions to improve daily functions.^11^ The LVP-FVQ can also be used to complement clinical evaluation when assessing self-reported functional vision.^11^ We agree with Gothwal *et al*.^11^ that the addition of items related to mobility would allow broader application of LVP-FVQ to individuals who have acceptable visual acuity yet advanced peripheral field loss.

The results from the present study suggest that involvement of the parents and school teachers will help to avoid bias in the outcomes. This observation has been confirmed using different questionnaires.^17^,^18^ For example, previous studies have incorporated questions to gauge the impact of visual impairment on children and their families resulting in a useful tool for the pediatric vision research community.^17^,^18^ Recently, Cochrane *et al*. (2008)^19^ studied the impact of vision impairment on participation in daily activities of school-aged children (8-18 years). Cochrane *et al*. considered this the primary step in developing a pediatric vision-related quality of life instrument.^19^ They used separate focus groups for students with low vision, parents and teachers, concluding that the perspective of the various stakeholders is crucial to the development of a new questionnaire.^19^ Such input will result in a balanced questionnaire.

Visual function assessment may vary between cultures, communities and countries consequently affecting the results. Other factors that affect outcomes are medical treatment and rehabilitation training. Results from the present study revealed some differences in visual function compared to Indian children using the same tool. The LVP-FVQ is a useful tool and may be used in assessment of visual function in Egyptian children with low vision. However, no association was found between different demographic and social characteristics and the level of severity in visual functioning. A major limitation of this study is its relatively small sample size. The other limitation is the need to transfer the raw score to a continuous variable. We believe this pilot study emphasizes the need for additional studies with a larger sample size, and modifications of the tool to include the input of parents and teachers which would further enhance the use of this method of assessment.
